# Muscæ Volitantes

**Published:** 1874-07

**Authors:** 


### MUSCÆ VOLITANTES.

The English of which is "floating flies," and consists in that difficulty of vision in which floating specks, of various shapes and hues, are seen before the eyes.

The specks take upon themselves the queerest fantastic shapes. Sometimes they resemble small, transparent disks or circles, arranging themselves into grotesque forms, sometimes seen single, and again in groups. Often their form is that of spiders, flies, and other insects. Again, delicate threads, resembling the spider's web, are seen, and the patient strives to brush the offending particle from before the eyes. At other times larger motes are seen, appearing as large dark spots, gradually extending over the field of vision until one-half the eye, or even more, is rendered perfectly blind.

There exists several causes for these symptoms, all more or less dangerous. Nervous, dyspeptic people, particularly women, who are much given to sewing, are troubled with dark or brilliant spots floating before their eyes. They become more apparent when the person regards some white or illuminated surface, when the spots appear dark. Looking upon a dark ground will give them a brilliant hue, resembling strings of bright beads. They are increased by fatigue or by derangement of the digestive apparatus.

Another sort are developed by injuries to the eye, as from a blow, when a small choroidal or retinal vessel is ruptured, and the blood escapes into the vitrious humor. The same thing may occur, however, where there is much congestion of the internal membranes of the eye, the vessels often rupturing through diseased action. Spots so produced are large and black, often completely obstructing vision. In a few days the blood begins to absorb, when the vision becomes partially clear, the particles taking on the appearance of hairs or irregular blotches. When these ruptures occur in the back portion of the eye, the retina is lifted from its position, and ever after remains more or less detached, giving rise to partial or complete blindness, depending on the amount of effusion, or the skillfulness of the attending oculist.

Spots or motes flying before the vision, are always the occasion of serious alarm. Sometimes they are of a nature that will never result in any serious impairment of vision; but most frequently they are indicative of the most serious lesions in the interior of the eye, which, when neglected, will result in permanent and incurable blindness. A skillful ophthalmic surgeon should at once be sought, who, by aid of the ophthalmoscope, can view the interior of the eye, seeing every portion of the darkened chamber as clearly as you can trace the lines upon your hand. By aid of this instrument the spots can be detected in the vitreous humor, or the effusion of blood discovered under the retina; or, if neither exists, the symptoms depending upon anæmia, congestion or abuse of the eyes, the ophthalmic surgeon can dismiss you with proper advice for their care, and you will be relieved of all anxiety upon the subject. In any case, never fail to learn their *cause;* for, as above intimated, they are the forerunners of serious disturbances to the visual organs.

